# Bone Density in Transgender Youth on Gender-Affirming Hormone Therapy

**DOI:** 10.1210/jendso/bvae045

**Published:** 2024-03-12

**Authors:** Micaela K Roy, Samantha Bothwell, Megan M Kelsey, Nina S Ma, Kerrie L Moreau, Kristen J Nadeau, Micol S Rothman, Natalie J Nokoff

**Affiliations:** Department of Pediatrics, University of Colorado School of Medicine, Aurora, CO 80045, USA; Department of Pediatrics, University of Colorado Anschutz Medical Campus, Aurora, CO 80045, USA; Department of Pediatrics, University of Colorado Anschutz Medical Campus, Aurora, CO 80045, USA; Department of Pediatrics, University of Colorado Anschutz Medical Campus, Aurora, CO 80045, USA; Department of Medicine, University of Colorado Anschutz Medical Campus, Aurora, CO 80045, USA; Geriatric Research Education and Clinical Center, Veterans Affairs Eastern Colorado, Aurora, CO 80045, USA; Department of Pediatrics, University of Colorado Anschutz Medical Campus, Aurora, CO 80045, USA; Department of Medicine, University of Colorado Anschutz Medical Campus, Aurora, CO 80045, USA; Department of Pediatrics, University of Colorado Anschutz Medical Campus, Aurora, CO 80045, USA

**Keywords:** transgender, bone, gender-affirming hormone therapy, testosterone, estradiol, gonadotropin-releasing hormone agonist

## Abstract

Some transgender youth are treated with gonadotropin-releasing hormone agonists (GnRHa) followed by testosterone or estradiol, which may impact bone mineral density (BMD). This cross-sectional study of transgender youth (n = 56, aged 10.4-19.8 years, 53% assigned female at birth [AFAB]) utilized total body dual-energy x-ray absorptiometry to evaluate BMD Z-scores, and associations between GnRHa duration, body mass index (BMI), and BMD. Participants on GnRHa alone (n = 19, 14 assigned male at birth [AMAB], 5 AFAB) at the time of the study visit were 13.8 [12.8, 15.3] (median [IQR]) years old, had been on GnRHa for 10 [5.5, 19.5] months, and began GnRHa at age 12 [10.4, 12.6] years. Total body BMD Z-score for individuals on GnRHa monotherapy was −0.10 [−0.8, 0.4] (AFAB, female norms) and −0.65 [−1.4, 0.22] (AMAB, male norms). AFAB participants (n = 21) on testosterone were age 16.7 [15.9, 17.8] years, had been on testosterone for 11 [7.3, 14.5] months, and started testosterone at age 16 [14.8, 16.8] years; total body BMD Z-score −0.2 [−0.5, 0] (male norms) and 0.4 [−0.2, 0.7] (female norms). AMAB participants (n = 16) were age 16.2 [15.1, 17.4] years, had been on estradiol for 11 [5.6, 13.7] months, and started estradiol at age 16 [14.4, 16.7] years; total body BMD Z-score −0.4 [−1.1, 0.3] (male norms) and −0.2 [−0.7, 0.6] (female norms). BMD Z-score was negatively correlated with GnRHa duration (male norms: *r* = −0.5, *P* = .005; female norms: *r* = −0.4, *P* = .029) and positively correlated with BMI (male norms: *r* = 0.4, *P* = .003; female norms: *r* = 0.4, *P* = .004). In this cross-sectional cohort, total body BMD Z-scores were slightly below average, but lowest in the AMAB group on GnRHa monotherapy.

About 1.4% of adolescents in the United States identify as transgender (gender identity differs from sex assigned at birth) [1]. Guidelines recommend initiation of gonadotropin-releasing hormone agonists (GnRHa) for eligible transgender youth after the onset of puberty (Tanner stage 2) to prevent development of secondary sex characteristics that do not align with gender identity [2, 3]. Some individuals will go on to start gender-affirming hormone therapy (GAHT) with testosterone or estradiol to induce secondary sex characteristics that align with gender identity [2, 3]. Not all individuals on testosterone or estradiol will have previously received GnRHa therapy, due to of age of presentation, insurance coverage, or other factors.

It is well-known that sex steroids regulate bone development, impact cortical bone expansion, maintain trabecular bone turnover, and contribute to the observed differences in bone mineral density (BMD) between people assigned male or female at birth (AMAB and AFAB, respectively) [4, 5]. Estradiol impacts bone development through the inhibition of sclerostin, promotion of osteoblasts, the suppression of osteoclast formation, the induction of osteoclast apoptosis [6], and closure of the epiphyseal growth plates [7]. Estradiol deficiency in both sexes results in declines in bone formation markers and BMD [6]. Testosterone is thought to affect bone development through direct action or through increases in muscle mass and decreases in body fat [8], although most of its effects are from aromatization to estradiol [9].

Bone health among transgender individuals is a topic of interest, given the multiple mechanisms by which GnRHa therapy, testosterone, and/or estradiol impact BMD and bone architecture [5]. Furthermore, recent studies show that baseline BMD is low among transgender youth, especially AMAB individuals, prior to any medical therapy [10, 11]. BMD Z-scores decline with GnRHa monotherapy but typically improve with testosterone or estradiol GAHT [11, 12], although areal BMD may remain low in some transgender women [12]. Many of the longitudinal studies are from Europe, where adolescents typically start GnRHa at an older age or later pubertal stage than in the United States. There are limited studies examining the effect of GnRHa treatment duration on BMD, or whether there are differences between individuals who did or did not receive GnRHa treatment before starting GAHT. To address current gaps in knowledge, we performed a secondary analysis to assess BMD in a cross-sectional study of transgender youth undergoing different GAHT and/or GnRHa treatment regimens. We aimed to evaluate overall BMD Z-scores and their associations with GnRHa treatment for youth on GnRHa alone or testosterone or estradiol +/− GnRHa.

## Methods

### Participants

Transgender youth (n = 56, age 10.4–19.8 years, 53% AFAB) who had been on GnRHa, testosterone, or estradiol for ≥ 3 months were recruited from Children's Hospital Colorado (2016–2018). This was a secondary analysis of a cross-sectional study evaluating insulin sensitivity and body composition [13, 14]. GnRHa were started at or after Tanner 2 pubertal development. Participants were excluded if they had significant medical or mental health comorbidities or were taking hormones not medically prescribed. No participants had known metabolic bone disease; past fracture data were not collected for the primary study. Consent or assent was obtained from all participants, and consent was obtained from a guardian (for those < 18 years old at enrollment). The study was approved by the Colorado Multiple Institutional Review Board.

### Research Visit

Visits and blood draws were performed at the Clinical Translational Research Center (CTRC) in the morning after an overnight fast. Testosterone and estradiol were measured by chemiluminescence (Beckman Coulter, Brea, CA, inter- and intra-assay coefficient of variations previously published [13, 14]). A single pediatric endocrinologist evaluated participant pubertal stage by assessing pubic hair, either breast development by inspection and palpation, or testicular volume using a Prader orchidometer. Tanner stage by testicular volume was defined as: Tanner 1, < 4 mL; Tanner 2, > 4 mL and < 8 mL; Tanner 3, ≥ 8 mL and < 12 mL; Tanner 4, ≥ 12 mL and ≤ 15 mL; and Tanner 5, > 15 mL [15]. Height was measured on a Harpenden stadiometer (recorded to 0.1 cm) and weight on a digital electronic scale (recorded to 0.1 kg) in light clothing. Body mass index (BMI) percentile was then calculated using the 2000 Centers for Disease Control and Prevention Growth Charts according to sex assigned at birth [16]. Participant demographic data was assessed with a questionnaire. Collected data were managed with Research Electronic Data Capture (REDCap) electronic data capture tools hosted at the University of Colorado Anschutz Medical Campus.

BMD was assessed with total body dual-energy x-ray absorptiometry (DXA, Discovery A, Hologic, Marlborough, MA). Median and interquartile ranges are presented. Group differences in BMD were evaluated with Mann-Whitney U tests (BMD data were nonparametric) and associations with Spearman correlations. BMD Z-scores were calculated for AMAB individuals on estradiol and AFAB individuals on testosterone using both male and female norms. Height for age Z-score adjusted BMD were also calculated for individuals on GnRHa monotherapy. Missing data were not included (2 participants did not have DXA).

## Results

Demographics and DXA results are in Table 1. Participants on GnRHa alone (n = 19, 5 AFAB, 14 AMAB) at the time of the study visit were aged 13.8 [12.8, 15.3] (median [IQR]) years, had been on GnRHa for 10 [5.5, 19.5] months before study enrollment, and began GnRHa treatment at age 12 [10.4, 12.6] years. AFAB participants (n = 21, 5 with past GnRHa) on testosterone were age 16.7 [15.9, 17.8] years, had been on testosterone for 11 [7.3, 14.5] months before study enrollment, and started testosterone at age 16.0 [14.8, 16.8] years. AMAB participants (n = 16, 6 with current or past GnRHa) were aged 16.2 [15.1, 17.4] years, had been on estradiol for 11 [5.6, 13.7] months before study enrollment, and started estradiol at age 16 [14.4, 16.7] years.

**Table 1.** Participant characteristics and DXA Z-scores

	All on GnRHa alone n = 19	AFAB on GnRHa alone n = 5	AMAB on GnRHa alone n = 14	AFAB on testosterone n = 21	AMAB on estradiol n = 16
Age, years	13.8 [12.8, 15.3]	13.5 [13.3, 14.4]	13.2 [11.5, 13.6]	16.7 [15.9, 17.8]	16.2 [15.1, 17.4]
Weight Z-score	−0.46 [−1.47, −0.53]	−0.78 [−1.33, −0.06]	−1.09 [−1.75, −0.3]	0.37 [−0.36, 0.72]	−0.23 [−0.82, 0.93]
Height Z-score	−0.59 [−1.49, −0.12]	−0.78 [−2.28, −0.20]	−0.68 [−1.32, −0.26]	0.0 [−0.79, 0.46]	−0.57 [−1.6, −0.28]
BMI Z-score	−0.28 [−1.33, 0.59]	−0.14 [−0.53, 0.19]	−0.92 [−1.87, 0.0]	0.52 [−0.60, 0.96]	0.28 [−0.92, 1.56]
Sex assigned at birth, n (%)					
Male	14 (74)	0 (0)	14 (100)	0 (0)	16 (100)
Female	5 (26)	5 (100)	0 (0)	21 (100)	0 (0)
Past or current GnRHa, n (%)	19 (100)	5 (100)	14 (100)	5 (24)	6 (38)
Duration of therapy, months	10 [5.5, 19.5]	10 [6, 22]	10 [5, 16.5]	11 [7.3, 14.5]	11 [5.6, 13.7]
Age at initiation of therapy, years	12 [10.4, 12.6]	12 [9.4, 12.4]	12 [10.6, 12.7]	16 [14.8, 16.8]	16 [14.4, 16.7]
Race n (%)					
White	15 (79)	3 (60)	12 (86)	15 (71)	14 (88)
Another race	4 (21)	2 (40)	2 (14)	6 (29)	2 (12)
Hispanic/Latino ethnicity n (%)	8 (42)	2 (40)	6 (43)	7 (33)	2 (13)
Breast/testicular stage n (%)					
1	0 (0)	0 (0)	0 (0)	0 (0)	1 (7)
2	8 (42)	1 (20)	7 (50)	0 (0)	1 (7)
3	4 (21)	2 (40)	2 (14)	0 (0)	2 (13)
4	1 (5)	0 (0)	1 (7)	0 (0)	1 (7)
5	3 (16)	0 (0)	3 (21)	19 (90)	3 (19)
Missing	3 (16)	2 (40)	1 (7)	2 (10)	8 (50)
Had menarche, n (%)	2 (40)	2 (40)	—	20 (95)	—
Age of menarche, years	12 [11.5, 12.5]	12 [11.5, 12.5]	—	12 [11.75, 13]	—
Cigarette smoking	1 (5)	1 (20)	0 (0)	3 (14)	3 (19)
# of days in the last week exercised > 20 min, enough to break a sweat					
0–2 days	5 (26)	2 (40)	3 (21)	11 (52)	10 (63)
3–5 days	9 (48)	2 (40)	7 (50)	6 (29)	4 (25)
6–7 days	5 (26)	1 (20)	4 (29)	4 (19)	2 (12)
BMD Z-Scores					
Total body BMD Z-score, male norms	−0.6 [−1.4, 0.3]	0.3 [−0.8, 0.3]	−0.7 [−1.4, 0.2]	−0.2 [−0.5, 0]	−0.4 [−1.1, 0.3]
Total height adjusted body BMD Z-score, male norms	−0.5 [−1.2, 0.2]	0.0 [−0.1, 0.4]	−0.6 [−1.2, 0.1]	—	—
Subtotal height adjusted body BMD Z-score, male norms	−0.7 [−1.3, −0.3]	0.0 [−0.2, 0.4]	−1.3 [−1.4, −0.5]	—	—
Total body BMD Z-score, female norms	−0.8 [−1.7, 0.2]	−0.1 [−0.8, 0.4]	−0.9 [−1.7, 0.1]	0.4 [−0.2, 0.7]	−0.2 [−0.7, 0.6]
Total height adjusted body BMD Z-score, female norms	−0.9 [−1.3, −0.1]	−0.1 [−0.6, 0.3]	−1.0 [−1.8, −0.2]	—	—
Subtotal height adjusted body BMD Z-score, female norms	−1.1 [−1.4, −0.5]	−0.1 [−0.8, −0.1]	−1.2 [−1.9, −0.7]	—	—

Values are n (%), mean ± SD, or median (25%, 75%ile).

Abbreviations: AFAB, assigned female at birth; AMAB, assigned male at birth; BMD, bone mineral density; GnRHa, gonadotropin-releasing hormone agonist.

### BMD in Transgender Adolescents

The median (25%, 75%ile) total body BMD Z-score for all AFAB transgender patients on GnRHa alone was 0.3 [−0.8, 0.3] compared with male norms, and −0.1 [−0.8, 0.4] compared with female norms by DXA (Table 1). The BMD Z-score for all AMAB patients on GnRHa alone was −0.7 [−1.4, 0.2] compared with male norms, and −0.9 [−1.7, 0.1] compared with female norms.

The BMD Z-score for AFAB individuals on testosterone was −0.2 [−0.5, 0.0] compared with male norms, and 0.4 [−0.2, 0.7] compared with female norms. AFAB individuals on testosterone wtih prior GnRHa had significanty lower BMD Z-scores using female norms than those on testosterone alone (-0.3 [-0.6, -0.2] vs. 0.7 [0.2, 0.8], *P =* .010), but no differences using male norms (*P* = .106).

The BMD Z-score for AMAB individuals on estradiol was −0.4 [−1.1, 0.3] compared with male norms, and −0.2 [−0.7, 0.6] compared with female norms. There were no significant differences in BMD Z-scores for AMAB individuals on estradiol +/  GnRHa.

**Figure 1.**
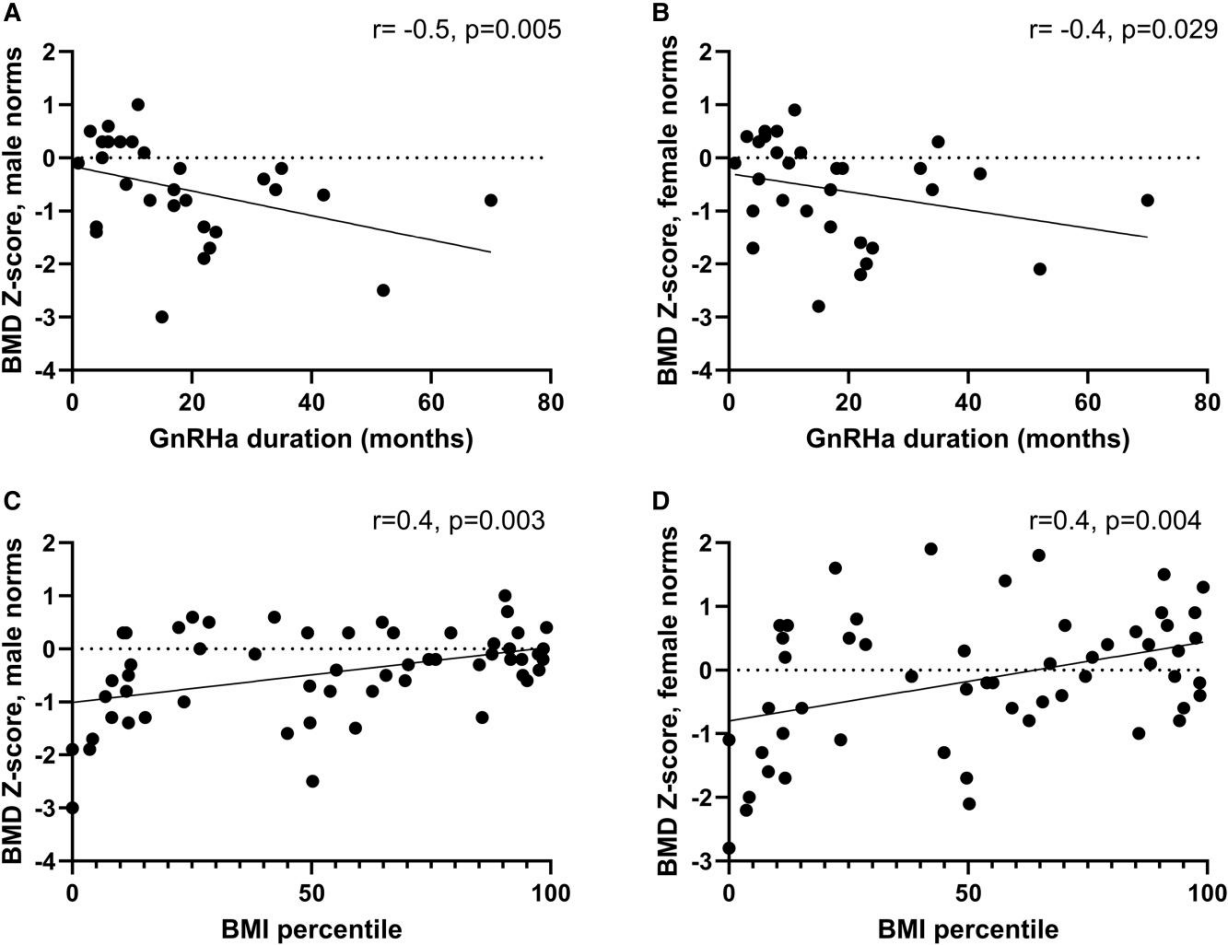
Variables significantly correlated with BMD Z-scores in transgender youth on GAHT. Duration of GnRHa therapy was negatively correlated with total body BMD Z-score with male norms (A, *r* = −0.5, *P* = .005) and female norms (B, *r* = −0.4, *P* = .029). BMI percentile was positively correlated with BMD Z-score with male norms (C, *r* = −0.4, *P* = .003) and female norms (D, *r* = 0.4, *P* = .004).

BMD Z-score correlations with GnRHa duration and BMI are in Fig. 1. BMD Z-score was negatively correlated with GnRHa duration (male norms: *r* = −0.5, *P* = .005; female norms: *r* = −0.4, *P* = .029). BMD Z-score was positively correlated with BMI (male norms: *r* = 0.4, *P* = .003; female norms: *r* = 0.4, *P* = .004). BMD Z-score was unrelated to duration of testosterone or estradiol therapy, sex steroid concentrations, or height Z-score.

## Discussion

In this cross-sectional cohort study of transgender youth on various GAHTs, total body BMD Z-scores were slightly below average. AMAB individuals on GnRHa monotherapy for a median of 10 months demonstrated the lowest BMD Z-scores within our cohort (although still in the normal range), and GnRHa duration negatively correlated with BMD.

Longitudinal studies demonstrate a decline in bone mineral apparent density Z-score and areal BMD after initiation of GnRHa, with improvement after initiation of testosterone [12] and mixed results for those starting estradiol [11, 12]. Recent studies show that BMD is low among transgender youth even prior to any medical therapy, particularly AMAB individuals[10, 11]. Our study was cross-sectional, not longitudinal, without data prior to initiation of GnRHa. Most of the data on BMD in transgender youth comes from Europe, where GnRHa and GAHT were generally started later in adolescence and at later pubertal stages (reviewed in [17]). In the United States, and as outlined in the Endocrine Society guidelines, GnRHa are initiated earlier, at Tanner stage 2 to 3 [2]. Studies of BMD in Tanner 2 to 3 transgender youth (reviewed in [17]) demonstrate low baseline BMD and decreases of BMD on GnRHa. Positive predictors of BMD Z-scores include sex (AFAB), vitamin D, and BMI-Z-score, and negative predictors include later age at GnRHa initiation [10]. Similarly, we showed a positive association between BMI and BMD Z-score in this cohort (with most individuals in the GnRHa group Tanner 2 to 3). We also showed a negative correlation of BMD Z-score with GnRHa duration. Longer-term studies are needed to determine if there is a maximum recommended duration of GnRHa monotherapy to better guide physicians, patients, and families.

Studies evaluating transgender adolescents in later puberty (Tanner 4 to 5) also show lower areal BMD and bone mineral apparent density Z-scores among AMAB individuals compared with AFAB individuals, decreases in BMD after GnRHa or progestin therapy, and mixed results after GAHT [17]. It is reassuring that the individuals in this study on testosterone or estradiol had normal BMD, although many did not have prior GnRHa treatment, and were later in puberty. Longitudinal studies show that initiation of testosterone or estradiol in transgender youth improves BMD, though full normalization of Z-scores in AMAB individuals on estradiol is not always demonstrated [11].

There are many factors aside from GnRHa and GAHT that may impact bone health in transgender youth, including low BMI, eating disorders [18], low physical activity, low vitamin D intake, and others [17]. In this study, we had a crude measure of physical activity, asking individuals how many days in the last week they exercised enough to break a sweat for 20 minutes or more. Up to a quarter of our youth exercised nearly every day, which may be higher than in other cohorts.

There are several limitations to this study. This was a small, cross-sectional study with no baseline data prior to starting GnRHa or GAHT. Data were collected at a single center and may not be generalizable to other sites or different patient populations. Physical activity was assessed with a single question, vitamin D was not measured, and fracture history was not assessed. Moreover, comorbid eating disorders were not screened for, which could impact the results. A significant proportion of AMAB individuals refused testicular Tanner staging. GnRHa therapy was started at a variety of ages and Tanner stages based on timing of presentation to clinical care. One participant had a testicular volume consistent with Tanner 1 at the time of the study but was Tanner 2 at initiation of GnRHa treatment (testicular size can regress somewhat after either GnRHa or estradiol). Although we corrected BMD Z-score for height in the GnRHa group, bone ages were not available to correct based on bone age (likely more relevant to the GnRHa monotherapy group). Despite its limitations, this study provides additional insights into the impact of duration of GnRHa therapy on BMD and the relation between BMD and BMI in this population.

In summary, total body BMD Z-scores ascertained by DXA were slightly below average for female and male norms, but still in the normal range, including for those who were on GnRHa monotherapy and normal for those on GAHT. GnRHa duration was negatively correlated and BMI positively correlated with BMD Z-score. Optimizing physical activity, BMI, and vitamin D status is important for this population. Newer studies are utilizing quantitative computed tomography to evaluate bone microarchitecture and strength, as well as evaluating bone marrow composition and markers of bone turnover that may provide additional insights [17]. More research is needed to determine the optimal length of GnRHa therapy and/or timing of GAHT initiation with testosterone or estradiol and to better counsel patients and families with regard to bone health outcomes.

## Data Availability

The datasets generated during and/or analyzed during the current study are not publicly available but are available from the corresponding author on reasonable request.

## Clinical Trial Information

Clinicaltrials.gov registration no. NCT02550431.

## Abbreviations

AFAB: assigned female at birth
AMAB: assigned male at birth
BMD: bone mineral density
BMI: body mass index
DXA: dual-energy x-ray absorptiometry
GAHT: gender-affirming hormone therapy
GnRHa: gonadotropin-releasing hormone agonist
